# Association of blood transfusion-related DEHP exposure with gut microbiota alterations in preterm infants

**DOI:** 10.1016/j.jped.2025.101476

**Published:** 2025-11-28

**Authors:** Yung-Ning Yang, Yu-Chen S.H. Yang, San-Nan Yang, Ying-Yu Chen, Hung-Yun Lin, Jau-Ling Suen

**Affiliations:** a I-Shou University, E-DA Hospital, Department of Pediatrics, Kaohsiung, Taiwan; b I-Shou University, College of Medicine, School of Medicine, Kaohsiung, Taiwan; c Taipei Medical University, Office of Human Research, Joint Biobank, Taipei, Taiwan; d Taipei Medical University, College of Nutrition, Graduate Institute of Metabolism and Obesity Sciences, Taipei, Taiwan; e Kaohsiung Medical University, College of Medicine, Graduate Institute of Medicine, Kaohsiung, Taiwan; f Taipei Medical University, College of Medical Science and Technology, Graduate Institute of Cancer Molecular Biology and Drug Discovery, Taipei, Taiwan; g Taipei Medical University, Wan Fang Hospital, Cancer Center, Taipei, Taiwan; h Pharmaceutical Research Institute, Albany College of Pharmacy and Health Sciences, Albany, USA; i Taipei Medical University, TMU Research Center of Cancer Translational Medicine, Taipei, Taiwan; j Taipei Medical University, Taipei Medical University Hospital, Traditional Herbal Medicine Research Center, Taipei, Taiwan; k Kaohsiung Medical University, Research Center for Precision Environmental Medicine, Kaohsiung, Taiwan; l Kaohsiung Medical University Hospital, Department of Medical Research, Kaohsiung, Taiwan

**Keywords:** Blood transfusion, Di-(2-ethylhexyl) phthalate, *Enterococcus*, Gut Microbiota

## Abstract

**Objective:**

Avoiding early-life exposure to environmental endocrine disruptors (EDCs) is critical for neonatal growth and long-term health. However, exposure to Di-(2-ethylhexyl) phthalate (DEHP), a typical EDC and plasticizer, is unavoidable in neonatal intensive care units due to contact with polyvinyl chloride medical devices.

**Methods:**

This study examined the impact of DEHP exposure through blood transfusion on gut microbiota development in preterm infants. To address this aim, the authors conducted a prospective cohort study, enrolling preterm infants between May 1, 2016, and March 30, 2021, who had not received blood transfusions or antibiotic treatment for at least seven consecutive days prior to enrollment. Fecal samples collected before and after transfusion underwent 16S ribosomal RNA gene-based next-generation sequencing analysis, while DEHP levels were measured in post-transfusion blood products. The average DEHP level in these products was 0.072 ± 0.03 mg/mL.

**Results:**

Initial analysis of 23 preterm infants (including five recruited twice) indicated that blood transfusion did not significantly alter bacterial composition and diversity. However, when examining recruitment events by postmenstrual age (PMA) or days of life (DOL), a significant increase in *Enterococcus* abundance was observed in both the lower PMA and younger DOL groups.

**Conclusions:**

These findings suggest a possible association between early-life DEHP exposure via blood transfusion and altered gut microbiota. Further studies are needed to clarify causality and long-term health implications.

## Introduction

Premature infants born before 37 weeks of gestational age and being cared for in neonatal intensive care units (NICUs) are particularly vulnerable to environmental endocrine disruptors (EDCs). One of these disruptors is di-(2-ethylhexyl) phthalate (DEHP), a plastic softener present in polyvinyl chloride (PVC) medical devices [1,2,3]. PVC-made medical devices include blood storage bags and a variety of medical tubing, such as nasogastric tubes, endotracheal tubes, and tubing for total parenteral nutrition [2,3].

Among the medical treatments in NICU, blood transfusions result in significantly higher DEHP exposure, up to 0.3 (0.14–0.72) mg DEHP/kg body weight (BW)/day, due to DEHP leaching from PVC devices and its lipophilic properties enhancing red blood cell stability [4]. Two main reasons are speculated: firstly, DEHP non-covalently binds to PVC-made medical devices, allowing it to leach during medical procedures [3]; and secondly, as DEHP is lipophilic, it can integrate with the membrane and increase the stability of red blood cells when they are stored in DEHP-based blood bags [5]. Recent biomonitoring data also demonstrate that DEHP exposure post-transfusion shows significantly higher urinary levels (191.4 μg/L) than parenteral nutrition (22.0 μg/L) or central lines (23.1 μg/L) [6]. Although there is no report about short-term toxicity under the Scientific Committee on Emerging and Newly-Identified Health Risks (SCENIHR) guidelines for DEHP exposure to premature infants, the impact of this iatrogenic DEHP exposure on these vulnerable populations is worthy of further investigation.

Accumulating studies have shown that prenatal or postnatal exposure to DEHP may adversely affect the reproductive [7,8], and nerve systems [9] and immune responses [10,11]. Epidemiological studies reveal the associations between DEHP early-life exposure and the risk of allergic development in children [10], suggesting that maternal DEHP exposure may modulate neonatal immunity. The recent transgenerational murine study demonstrates that maternal DEHP chronic exposure at an environmentally relevant dose (0.037 mg DEHP/kg BW/day) alters offspring dendritic cell homeostasis through epigenetic modification [11]. These studies reveal that maternal exposure to DEHP in daily life at environmental doses modulates neonatal immunity development, making offspring more prone to childhood allergies; however, whether high-dose and short-term exposure to DEHP via blood transfusion impacts the immune development of newborns warrants further investigation.

Early-life gut microbiota dysbiosis is linked to immune-related disorders in children [12,13]. Studies show that lower levels of *Faecalibacterium, Lachnospira, Veillonella*, and *Rothia* increase allergy risks [13], while higher *Escherichia coli* and *Klebsiella pneumonia* levels are associated with atopic dermatitis [14]. In addition, a portion of the initial gut microbiota of newborn infants is acquired through maternal transmission from the amniotic fluid and placenta before birth [15]. With weekly gut microbiota analysis spanning postmenstrual age (PMA) 24 to 46 weeks, preterm infants’ microbiota can be divided into three phases, and the delayed phase transition appears to be associated with variation in nutritional intake and growth failure in infants [16]. The authors' previous study showed that early-life DEHP exposure significantly altered gut microbiota and anti-HBsAg-IgM response in neonates receiving intravenous fluid [17]. These studies suggest early-life EDC exposure, such as DEHP release in NICU, might impact newborn gut microbiota development.

In this prospective cohort study, the authors aimed to investigate whether high-dose DEHP exposure through blood transfusion is associated with alterations in gut microbiota composition in preterm infants. Given that PMA and days of life (DOL; equivalent to postnatal age) may influence the developmental vulnerability of the gut microbiota, the authors further explored whether the association varies across different developmental stages. This study was designed to provide insights into how iatrogenic DEHP exposure might affect early-life gut microbiota development in this vulnerable population.

## Patients and methods

### Study subject enrollment

From May 1, 2016, to March 30, 2021, a prospective cohort study (EMRP47104N) was conducted at the NICU of E-Da Hospital, enrolling premature infants (gestational age < 37 weeks) who had not received blood transfusions or antibiotics for at least seven consecutive days prior to enrollment. The study was approved by the Institutional Review Board and conducted in accordance with the Declaration of Helsinki. Infants with necrotizing enterocolitis, spontaneous intestinal perforation, or other gastrointestinal pathology were excluded.

All enrolled infants received mixed enteral feeding (breast milk and formula) and daily probiotic supplementation (*Lactobacillus reuteri* DSM 17,938, BioGaia, Stockholm, Sweden; 1 × 10⁸ CFU). However, the specific proportions and timing of milk types were not recorded, and no infant received exclusive feeding.

When hemoglobin levels fell below 11 g/dL and transfusion was indicated, irradiated packed red blood cells (20 mL/kg body weight) stored in DEHP-containing PVC bags (JMS Blood Bag, JMS Co., Ltd., Hiroshima, Japan) were administered. Fecal samples were collected at standardized time points: 1–2 days before transfusion (T0), and on days 1, 3, or 6 post-transfusion (T1, T2, T3). All samples were collected using sterile techniques and stored at –80 °C for 16S ribosomal RNA (rRNA)-based next-generation sequencing (NGS) analysis.

### Detection of DEHP levels in blood products

DEHP levels were measured in blood samples from 10 transfusion events. These samples were obtained from the remaining blood in transfusion bags after clinical use. Because not all transfusion events had sufficient residual volume for analysis, only 10 events were included. To simulate the actual exposure pathway in neonates, blood from each unit was passed through the standard PVC transfusion tubing before collection. The DEHP concentration was analyzed using LC–MS/MS. After appropriate sample preparation, 10 μL of the processed blood sample was injected into the instrument via the autosampler. The estimated DEHP dose per transfusion (mg/kg BW) was calculated using the following formula: DEHP concentration (mg/mL) × 20 mL/kg BW. Detailed analytical procedures are provided in the Supplementary Methods.

### Stool DNA extraction and 16S rRNA gene-based NGS sequencing

The procedures for extracting DNA from stool, constructing a library, and sequencing the V3-V4 region of bacterial 16S rRNA genes using an Illumina Miseq sequencer were carried out in accordance with the steps outlined in the Supplemental methods. The sequencing data has been deposited in the NCBI SRA under BioProject ID PRJNA1354601.

### Statistical analysis

Statistical analyses were performed using SPSS version 17.0 (SPSS Inc., Chicago, IL, USA). Data are presented as mean ± standard deviation (SD). Statistical differences were determined by using one-way ANOVA followed by a Bonferroni *t*-test for *post-hoc* multiple comparisons. A statistical significance level of *p* < 0.05 was applied to all tests.

To explore potential age-related patterns, recruitment events were stratified by the median PMA (240 days; equivalent to approximately 34.3 weeks) and by the median DOL (34 days; equivalent to approximately 4.9 weeks). These subgroup analyses were conducted as exploratory, post hoc evaluations, and the resulting p values were interpreted descriptively.

For time-course and temporal trend analyses, both PMA and DOL were converted to days. PMA was calculated as the sum of gestational ages (in days) at birth and the number of postnatal days (DOL) at the time of blood transfusion. This standardization enabled more accurate temporal comparisons of gut microbiota changes across infants with varying gestational ages at birth.

## Results

### Preterm infants who received blood transfusions were exposed to a high level of DEHP

The study initially enrolled 26 preterm infants. As 9 of them underwent two transfusion episodes, a total of 35 recruitment events were recorded. After excluding 7 events due to incomplete data, the final analysis included 28 transfusion-related recruitments from 23 infants, including 5 who contributed two events each (Figure S1). Blood transfusions were administered to infants with anemia to enhance oxygen delivery and stabilize vital signs [18]. In each recruitment, each enrolled infant contributed fecal samples one or two days before blood transfusion (defined as T0) and one, three, or six days after blood transfusion (T1, T2, and T3, respectively). The time points were chosen based on the known metabolic characteristics of DEHP in humans. After exposure, DEHP is rapidly metabolized, and peak levels of urinary metabolites are usually observed within a few hours, with most of them excreted within 24 h [19]. However, depending on the exposure level and individual variation, some metabolites may remain detectable for several days. Therefore, the authors collected fecal samples on days 1, 3, and 6 after transfusion to observe both the early and short-term effects on gut microbiota.

The clinical characteristics of these infants are summarized as follows. The average birth weight and gestational age of these infants were 1370.7 ± 737.4 g and 29.6 ± 3.3 weeks, respectively (Supplementary Table 1). To estimate DEHP exposure, residual blood remaining in the transfusion bags after administration was analyzed. The average DEHP concentration in these samples was 0.072 ± 0.03 mg/mL (Supplementary Table 2). Based on this concentration, the authors estimated that infants receiving a standard transfusion volume of 20 mL/kg BW were exposed to approximately 1.44 ± 0.6 mg/kg BW of DEHP. This exposure level substantially exceeds both the tolerable daily intake (TDI) for humans of 0.05 mg/kg BW/day [20] and the benchmark level of 0.3 mg/kg BW for a single transfusion [3], indicating a considerable exposure associated with blood transfusions in infants.

### Without age stratification, blood transfusion did not show an evident impact on the gut microbiota in the newborns

Fecal samples from 28 recruitment events were collected at four different times surrounding transfusion and analyzed for gut microbiota using 16S rRNA gene-based NGS sequencing. The study assessed alpha diversity through the Observed, Chao1, Shannon, and Simpson indices, and beta diversity via weighted UniFrac distances for PCoA. Initial analysis of these events indicated no statistically significant changes in gut microbiota composition before and after blood transfusion (Figure S2).

### Blood transfusion significantly increased intestinal enterococci at the genus level in the low PMA newborns

The authors categorized recruitment events by the median of PMA or DOL to tackle variations in preterm infant treatment and analysis. This approach is supported by research indicating that PMA, a measure combining gestational age at birth with chronological age post-birth, significantly influences the development of preterm infants' gut microbiota [16,21,22].

The authors divided the recruitment events into groups based on PMA median and investigated the effects of blood transfusion on the gut microbiota in infants with high or low PMA. The findings revealed that infants in the low PMA group (< 240 days; equivalent to approximately 34.3 weeks) had significantly lower birth weight, body weight at transfusion time, and gestational age compared to those in the high PMA group (≥ 240 days), with no differences in DOL or other factors such as Apgar scores, gender, and birth mode (Table 1).

**Table 1:** Characteristics of the preterm infants categorized by postmenstrual age (PMA) at the time of blood transfusion.

	Mean ± SD (min, max) or N		
Recruitment events *N* = 28	PMA < 240 days *N* = 14	PMA ≥ 240 days *N* = 14	*p* value
Birth weight (g)	1012.9 ± 273.6 (770, 1705)	1583.6 ± 855.2 (500, 3990)	0.022*
Body weight at the time of transfusion (g)	1350.0 ± 201.7 (966, 1656)	2689.2 ± 779.0 (1695, 4820)	< 0.001***
Gestational age at birth (day)^1^	192.1 ± 13.6 (175, 222)	214.9 ± 23.7 (175, 261)	0.010*
PMA at the time of transfusion (day)	225.9 ± 7.1 (211, 239)	266.7 ± 32.5 (240, 366)	< 0.001***
DOL at the time of blood transfusion (day)	33.8 ± 13.2 (11, 54)	51.9 ± 40.1 (14, 171)	0.213
Apgar1	5.8 ± 1.3	5.1 ± 2.8	0.845
Apgar5	7.9 ± 0.6	6.2 ± 3.2	0.227
Gender Boy Girl	7 7	10 4	0.440
Vaginal delivery	6	7	1.000

1 Gestational age in weeks: 192.1 days ≈ 27.4 weeks; 214.9 days ≈ 30.7 weeks.

**Figure 1:**
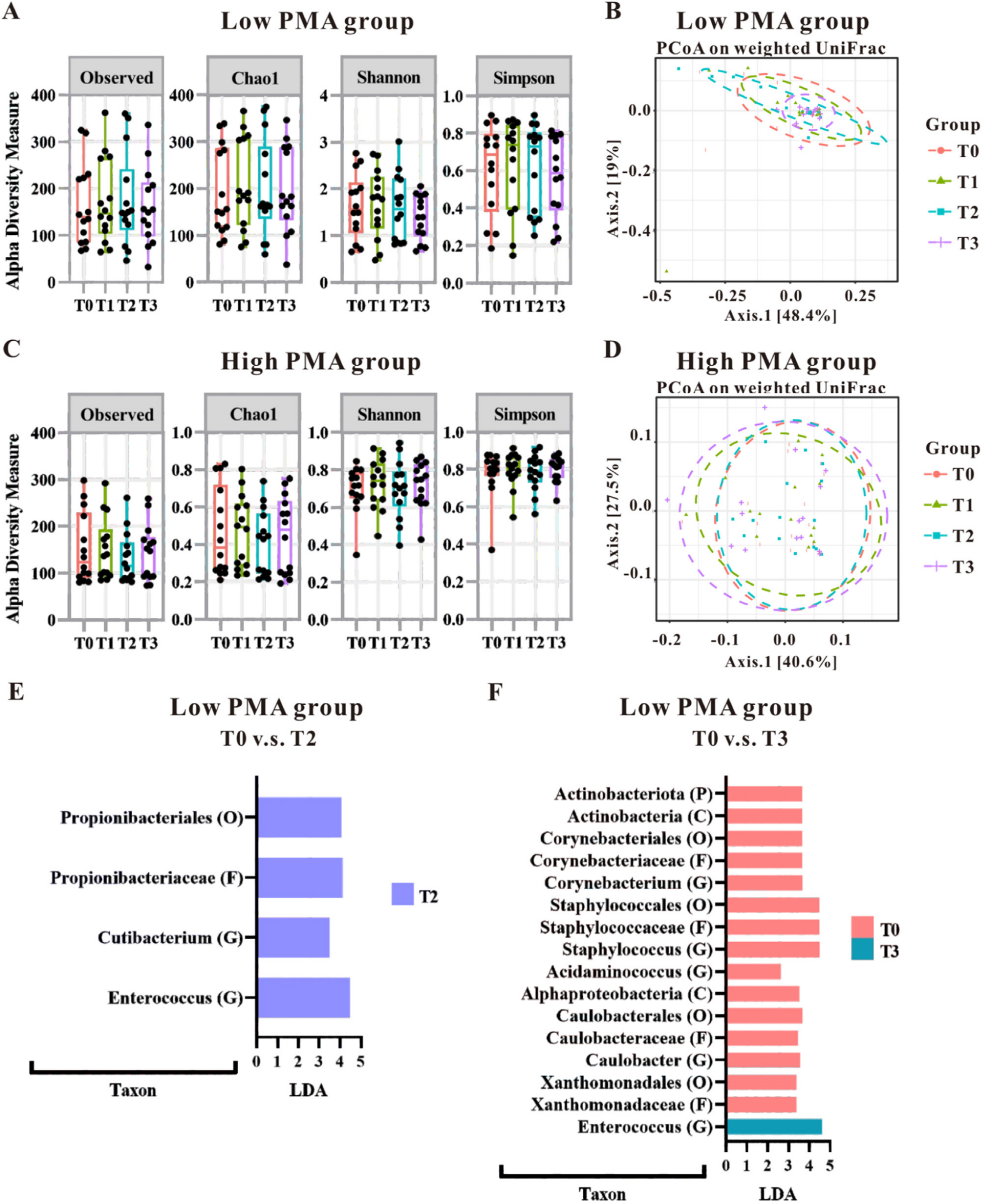
Gut microbial alteration of the preterm infants categorized by postmenstrual age (PMA) at the time of blood transfusion. The impact of transfusions on microbiota patterns was assessed in both low PMA groups (A and B) and high PMA groups (C and D) using alpha-diversity measures (A and C) and beta-diversity measures (B and D). The microbial taxonomy based on the 16S rRNA gene-based NGS sequencing was analyzed using LEfSe algorithm. LDA analysis was conducted to compare the low PMA group at T0 versus T2 (E), as well as T0 versus T3 (F). Taxa that achieved an LDA score greater than 2.0 were identified and labeled accordingly. Taxonomic levels ranging from phylum to species exhibited an LDA score greater than 2.0.

Initial analyses using alpha diversity and weighted UniFrac distances with PCoA indicated that gut microbiota patterns were not significantly altered by PMA levels (Figure 1A–1D); However, linear discriminant analysis (LDA) highlighted a significant impact of blood transfusion on the gut microbiota in the low PMA group (Figure 1E and 1F), with no notable effects in the high PMA group (data not shown). Specifically, in the low PMA group, blood transfusion led to increased levels of certain microbiota (*Propionibacteriales* at Order level, *Propionibacteriaceae* at Family level, and *Cutibacterium* and *Enterococcus* at Genus level) when comparing the T0 and T2 groups (Figure 1E). Further comparisons between the T0 and T3 groups revealed increased richness of *Enterococcus* in T3, while several microbial taxa showed increased richness in T0 (Figure 1D), including *Actinobacteriota* at Phylum level, *Actinobacteria* and *Alphaproteobacteria* at Class level, *Corynebacteriales, Staphylococcales, Caulobacterles* and *Xanthomonadales* at Order level, *Corynebacteriaceae, Staphylococcaceae, Caulobacteraceae* and *Xanthomonadaceae* at Family level and *Corynebacterium, Staphylococcus, Acidaminococcus* and *Caulobacter* at Genus level Notably, an increase in *Enterococcus* at the genus level was consistently observed in the low PMA group post-transfusion, underscoring a specific microbial change associated with blood transfusion in this demographic.

### Blood transfusion effects on gut microbiota was predominantly observed in the younger DOL group

**Figure 2:**
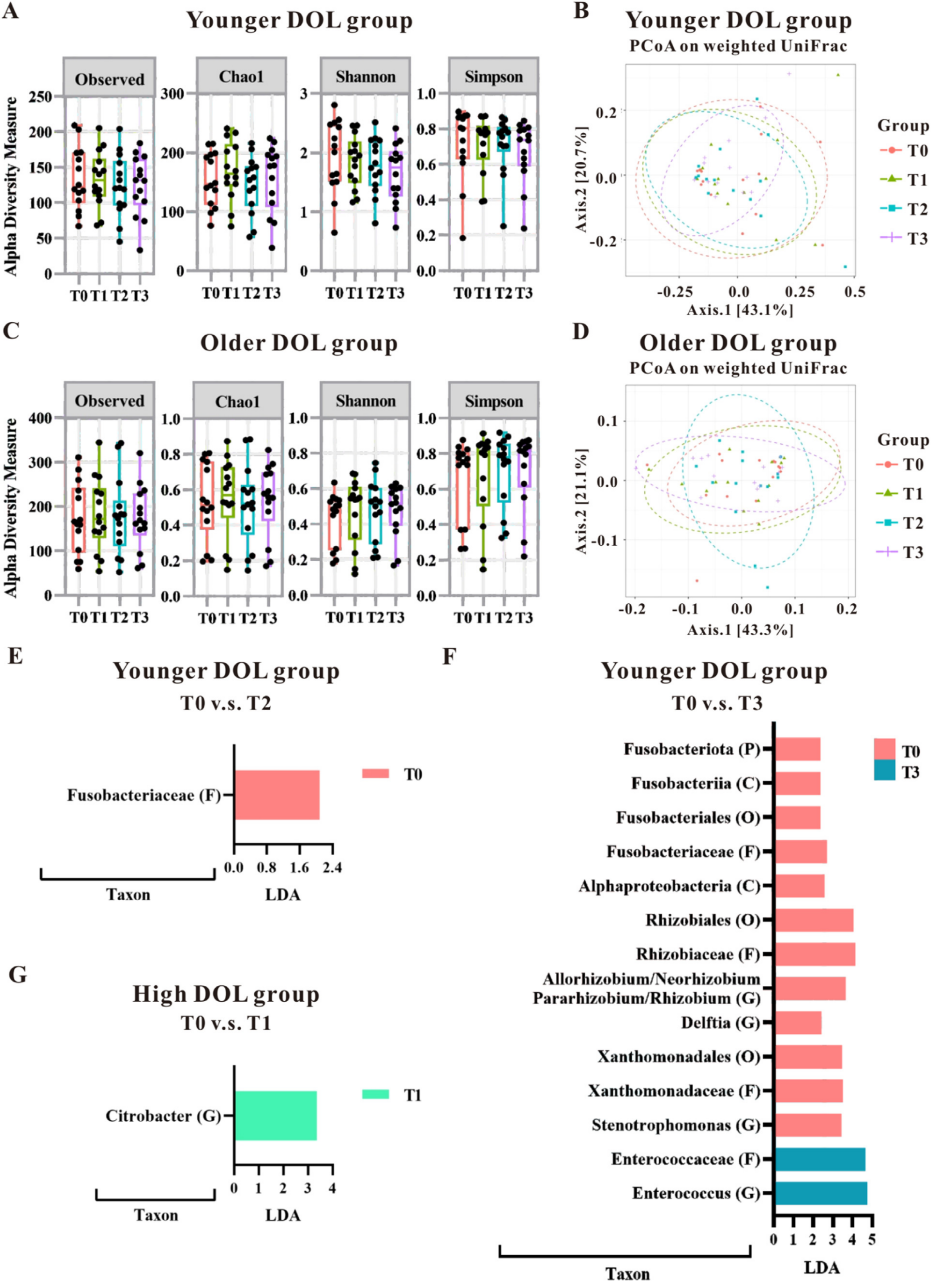
The gut microbial changes observed in preterm infants undergoing blood transfusions, categorized by day of life (DOL; equivalent to postnatal age) at the time of the transfusion. The impact of transfusions on microbiota patterns was evaluated in younger DOL groups (A and B) as well as older DOL groups (C and D) using alpha-diversity measures (A and C) and beta-diversity measures (B and D). Fecal samples were analyzed using the LEfSe algorithm to identify microbial taxonomy based on the 16S rRNA gene-based NGS sequencing. LDA analysis was conducted to compare the younger DOL group at T0 versus T2 (E), T0 versus T3 (F), and compare the older DOL group at T0 versus T1 (G). Taxa with an LDA score greater than 2.0 were identified and labeled accordingly. Taxonomic levels ranging from phylum to species exhibited an LDA score greater than 2.0.

The authors further analyzed the impact of blood transfusion on the groups categorized by the median of DOL. As shown in Table 2, the younger DOL group (< 34 days; equivalent to approximately 4.9 weeks) had significantly higher birth weight and gestational age than the older DOL group (≥ 34 days); however, there was no difference in PMA and body weight at the time of transfusion between these two groups. This suggests that although the infants differed in gestational age at birth and birth weight, their transfusions were given at comparable growth and developmental stages. The analysis of α-diversity and β-diversity showed that blood transfusion did not have a significant impact on the gut microbiota pattern based on DOL (Figure 2A–2D); however, the LDA analysis pinpointed specific microbial changes, particularly in the younger DOL group. Specifically, in the younger DOL group, a comparison of microbial changes between before (T0) and after (T2) blood transfusion revealed a significant decrease in the abundance of *Fusobacteriaceae* at the Family level at T2 (Figure 2E). Additionally, a comparison between T0 and T3 in the younger DOL group showed significantly high abundance in T0, including *Fusobacteriota* at Phylum level, *Fusobacteriia, Alphaproteobacteria* at Class level, *Fusobacteriales, Rhizobiales* and *Xanthomonadales* at Order level, *Fusobacteriaceae, Rhizobiaceae* and *Xanthomonadaceae* at Family level and *Allorhizobium*/*Neorhizobium*/*Pararhizbium*/*Rhizbium, Delftia* and *Stenotrophomonas* at Genus level (Figure 2F). However, the abundance of *Enterococcaceae* at Family level and *Enterococcus* at Genus level were significantly increased in T3 compared to T0 (Figure 2F). The LDA analysis revealed a single change in the older DOL group, where the abundance of *Citrobacter* at the Genus level was observed to increase at T1 compared to T0 (Figure 2G). These findings underscore that blood transfusions primarily affect the gut microbiota in infants with younger DOL, suggesting that transfusions may influence the developing gut microbiota in a stage-dependent manner.

**Table 2:** Characteristics of the preterm infants categorized by days of life (DOL) at the time of blood transfusion.

	Mean ± SD (min, max) or N		
Recruitment events *N* = 28	DOL < 34 days *N* = 14	DOL ≥ 34 days *N* = 14	*p* value
Birth weight (g)	1612.3 ± 827.3 (824, 3990)	984.1 ± 291.2 (500, 1440)	0.003**
Body weight at the time of transfusion (g)	2042.1 ± 1073.6 (966, 4820)	1997.1 ± 678.0 (1236, 3000)	0.635
Gestational age at birth (day)^1^	214.7 ± 21.9 (181, 261)	192.2 ± 16.6 (175, 231)	0.004**
PMA at the time of transfusion (day)	239.4 ± 23.4 (211, 293)	253.2 ± 36.8 (223, 366)	0.197
DOL at the time of transfusion (day)	24.6 ± 6.5 (11, 32)	61.0 ± 34.7 (34, 171)	< 0.001***
Apgar1	5.7 ± 2.2	5.2 ± 2.1	0.409
Apgar5	7.5 ± 2.0	6.6 ± 2.7	0.301
Gender Boy Girl	10 4	7 7	0.440
Vaginal delivery	7	6	1.000

1 Gestational age in weeks: 214.7 days ≈ 30.7 weeks; 192.2 days ≈ 27.5 weeks.

## Discussion

High-dose DEHP exposure during blood transfusions in premature infants, particularly those with lower PMA or younger DOL, significantly alters gut microbiota by increasing *Enterococcus* abundance, a pathogen causing urinary tract infections, meningitis, and sepsis in the NICU setting [23]. This change suggests a shift towards microbiota immaturity and potential adverse outcomes. The prevalence of *Enterococcus*, influenced by factors like diet and age, typically decreases with PMA [22,24,25]. However, these findings highlight an unexpected rise in post-DEHP exposure, implicating a potential risk for infections and underscoring the need for cautious DEHP use in neonatal care.

The authors’ study prior to this one found that full-term infants exposed to low doses of DEHP (approximately 0.001 to 0.027 mg/kg BW) through intravenous infusion, below the TDI of 0.05 mg/kg BW/day, experienced changes in their gut microbiota diversity and composition [20]. In contrast, premature infants exposed to a higher dose of DEHP (approximately 1.44 mg/kg BW) via blood transfusion showed no diversity changes but an increase in specific bacteria like *Enterococcus*. These results suggest that DEHP exposure, regardless of dose, may affect gut microbiota development, warranting further research on its health implications.

The development of gut microbiota is a vital factor contributing to the health of preterm infants [26], and it is associated with both gestational age [21] and PMA [16]. The current study observed that blood transfusion did have a significant impact on the gut microbiota composition of two groups of preterm infants. Infants with low gestational age showed an increased abundance of *Enterococcus* at the genus level three and six days after transfusion. Meanwhile, those with even higher gestational ages but fewer days since birth exhibited a similar increase in *Enterococcus*, but notably six days post-transfusion. This may suggest that preterm infants, particularly those younger in gestational age or with fewer days post-birth, could be more affected by EDCs like DEHP exposure in transfusions, highlighting the influence of gestational and postnatal age on their gut microbiota's response to environmental factors.

In Taiwan, critically ill premature infants in NICUs commonly show elevated DEHP metabolite levels due to extensive use of PVC-based medical devices such as tubing, bags, and respiratory equipment [27]. However, accurate estimation of DEHP exposure through urinary metabolites is challenging in preterm infants because of their immature renal function and unreliable creatinine-based correction [28]. To address this, the present study quantified DEHP levels directly from residual blood samples and calculated total exposure from transfusion volumes (Supplementary Table 2).

Although potential DEHP sources such as endotracheal tubes, feeding tubes, or intravenous lines exist [27,29], the authors carefully minimized their impact in this study. None of the enrolled infants were intubated, and all were fed using DEHP-free feeding tubes and received parenteral nutrition via DEHP-free bags (“Baxter” Exacta-Mix EVA Container). Thus, the blood transfusion procedure was the only major controlled and quantified source of DEHP exposure in this cohort. Besides, the prior findings indicated that DEHP exposure from 0.001 to 0.027 mg/kg BW/day in newborns not undergoing invasive procedures [17], emphasizing blood transfusions as a significant, high-dose DEHP exposure source for hospitalized preterm infants.

While the present study observed a temporal association between high-dose DEHP exposure through blood transfusion and an increase in *Enterococcus* abundance in preterm infants, the authors acknowledge that causality cannot be conclusively established. Direct measurements of serum or urinary DEHP metabolites, as well as inflammatory markers such as cytokines or hormones, were not performed. Therefore, other transfusion-related factors, such as anemia correction, intestinal reperfusion, transfusion volume, and increased iron load, may also have contributed to the observed microbial changes [30, 31, 32].

In the present study, all infants received standardized probiotics and were free from antibiotics or transfusions for at least seven days before enrollment. However, detailed data on feeding composition and schedule were lacking, which may have introduced residual confounding. Although the authors excluded infants with NEC or spontaneous intestinal perforation, transfusion-related NEC remains a concern. Future studies should include comprehensive clinical metadata and functional assays (e.g., metabolomics, cytokines) to clarify the role of DEHP and other transfusion-related factors in shaping gut microbiota and potential disease risk.

## Conclusions

This study highlights the potential alterations in gut microbiota, notably increased *Enterococcus*, associated with blood transfusion and the accompanying exposure to DEHP in preterm infants, despite its limitations, like small sample size and unavoidable plasticizer exposure in NICU settings. It underscores the need for further research into long-term effects and the development of DEHP-free medical materials.

## Abbreviations

BW, Body weight; DEHP, Di-(2-ethylhexyl) phthalate; DOL, Days of life; EDC, Environmental endocrine disruptor; LDA, Linear discriminant analysis; NGS, Next-generation sequencing; NICU, Neonatal intensive care unit; PMA, Postmenstrual age; PVC, Polyvinyl chloride; rRNA, Ribosomal RNA gene; TDI, Tolerable daily intake.

## Authors’ contributions

Yung-Ning Yang participated in the conception and design of the study, analysis and interpretation of data, drafting the article, and revising it critically for important intellectual content; Yu-Chen S.H. Yang participated in the conception and design of the study, data acquisition, analysis and interpretation; San-Nan Yang and Hung-Yun Lin participated in the conception and design of the study; Ying-Yu Chen participated in acquisition of data; Jau-Ling Suen participated in the conception and design of the study, drafting the article, revising it critically for important intellectual content and final approval of the version to be submitted.

## Funding

This work was supported by the 10.13039/501100008783National Science of Technology Council (MOST108–2314-B-214-010-MY2, MOST111–2314-B-214-007, NSTC 112–2320-B-037-031-MY3), 10.13039/501100004738E-Da Hospital (EDAHI111002, EDAHP112024, EDPJ111068, EDAHS113045), and the Kaohsiung Medical University Research Foundation (KMU-M110006). Additional support was provided by the Research Center for Precision Environmental Medicine, Kaohsiung Medical University, and TMU Research Center of Cancer Translational Medicine under the Featured Areas Research Center Program within the framework of the Higher Education Sprout Project by the Ministry of Education in Taiwan, as well as the Kaohsiung Medical University Research Center Grant (KMU-TC113A01). The authors also thank the TMU Core Laboratory of Human Microbiome for technological and analytical support.

## Data availability statement

The 16S rRNA gene sequencing data supporting the findings of this study have been deposited in the NCBI Sequence Read Archive (SRA) under BioProject accession number: PRJNA1354601.

## Conflicts of interest

The authors declare no conflicts of interest.

## Appendix Supplementary materials

Image, application 1
