# Supplementary material for: Association of blood transfusion-related DEHP exposure with gut microbiota alterations in preterm infants
Source: J Pediatr (Rio J). 2025 Nov 28;102(1):101476. doi: 10.1016/j.jped.2025.101476 (PMC12704293; doi:10.1016/j.jped.2025.101476)
Supplement: Supplementary file 1 [file mmc1.docx]

**JPED-D-25-00226_Supplementary Material**

**Association of Blood Transfusion-Related DEHP Exposure with Gut Microbiota Alterations in Preterm Infants**

**Supplementary information**

**Supplementary materials and methods**

**Detection of DEHP levels in blood products**

The detection and quantification of DEHP were conducted using a Waters ACQUITY UPLC system (Waters Corporation, Milford, MA) coupled with a Finnigan TSQ Quantum Ultra triple-quadrupole MS (Thermo Electron, San Jose, CA), assisted by the Xcalibur software (ThermoFinnigan, Bellefonte, PA). The LC-MS-MS system, equipped with an electrospray ion source (ESI), operated in positive mode. The sample injection volume was 10 μL, and the system featured an ACQUITY UPLC CSH Phenyl-Hexyl Column (130Å, 1.7 µm, 2.1 mm X 100 mm, Waters Corporation, Milford, MA), supplemented by a filter (Waters Acquity UPLC™ BEH C18 column, 1.7 μm, 2.1 mm × 5 mm) ahead of the column. With a flow rate of 250 μL/min and column temperature maintained at 40°C, the solvents used were A: 0.1% acetic acid in water and B: 0.1% acetic acid in acetonitrile. The solvent programming was set as follows: 0.0–1.0min, 25% B; 2.0–8.0min, 50% B; 10.0 min, 60% B; 11.0–13.0min, 80% B; 14.0–17.0min, 100% B; 17.5–20.0 min, 25% B. The settings for the MS-MS interphase were: spray voltage, 3000 V; sheath gas (N2) pressure, 28 psi; auxiliary gas (N2) pressure, 10 psi; capillary temperature, 350°C; collision gas (Ar) pressure, 1.0 mTorr. The standard protocol was used to determine the precursor/product ions in negative mode [M-H]–, the retention time of the analytes separated on the LC column, the individual collision energies, and the individual tube lens for the formation of product ions.

**Stool DNA extraction and 16S rRNA gene-based NGS sequencing**

The methodology for 16S rRNA gene-based sequencing and its analysis, thoroughly elaborated in Yang et al. 2020,[1] is briefly described here. Total genomic DNA extraction from fecal samples was carried out using a QIAamp Fast DNA Stool Mini Kit (QIAGEN, Germany). The authors followed the 16S rRNA Gene Amplicons protocol for the Illumina MiSeq System to prepare the library. Cutadapt (v 1.12) removed the gene-specific sequences targeted at the 16S V3 and V4 region from the demultiplexed paired reads. The authors utilized the R package DADA2 (v 1.14.1) in the R environment (v 3.6.1) to process the filtered reads, as described in Callahan et al. 2016.[2] The SILVA database (v138)[3] served as the reference for taxonomy assignment with a minimum bootstrap confidence of 80. DECIPHER (v2.14.0) was used for multiple sequence alignment of the SVs, and the phylogenetic tree was constructed from the alignment using phangorn (v2.5.5).[4] The authors integrated the count table, taxonomy assignment results, and phylogenetic tree into a phyloseq object, and conducted community analyses using phyloseq (v1.30.0).[5] The estimate_richness function from the phyloseq package was utilized to calculate the alpha-diversity indices. A statistical comparison was performed between pre- and post-transfusion samples, with an exact alpha set at 0.05 (using Kruskal-Wallis and Wilcoxon tests). The GUniFrac package (v1.1) was used to calculate UniFrac distances, enabling us to assess the community dissimilarity between groups.[6] Principal coordinate analysis (PCoA) ordination on UniFrac distances was executed, and the adonis and betadisper functions from the vegan package (v2.5.6) were used for statistical analysis to assess the dissimilarity of composition among groups and the homogeneity of dispersion respectively. The authors employed the Linear Discriminant Analysis (LDA) Effect Size (LEfSe) method for enrichment analysis between groups, applying the Wilcoxon-Mann-Whitney test (at α = 0.05) and considering a logarithmic LDA score greater than 2.[7] The results were visualized as a cladogram using GraPhlAn.[8]

**
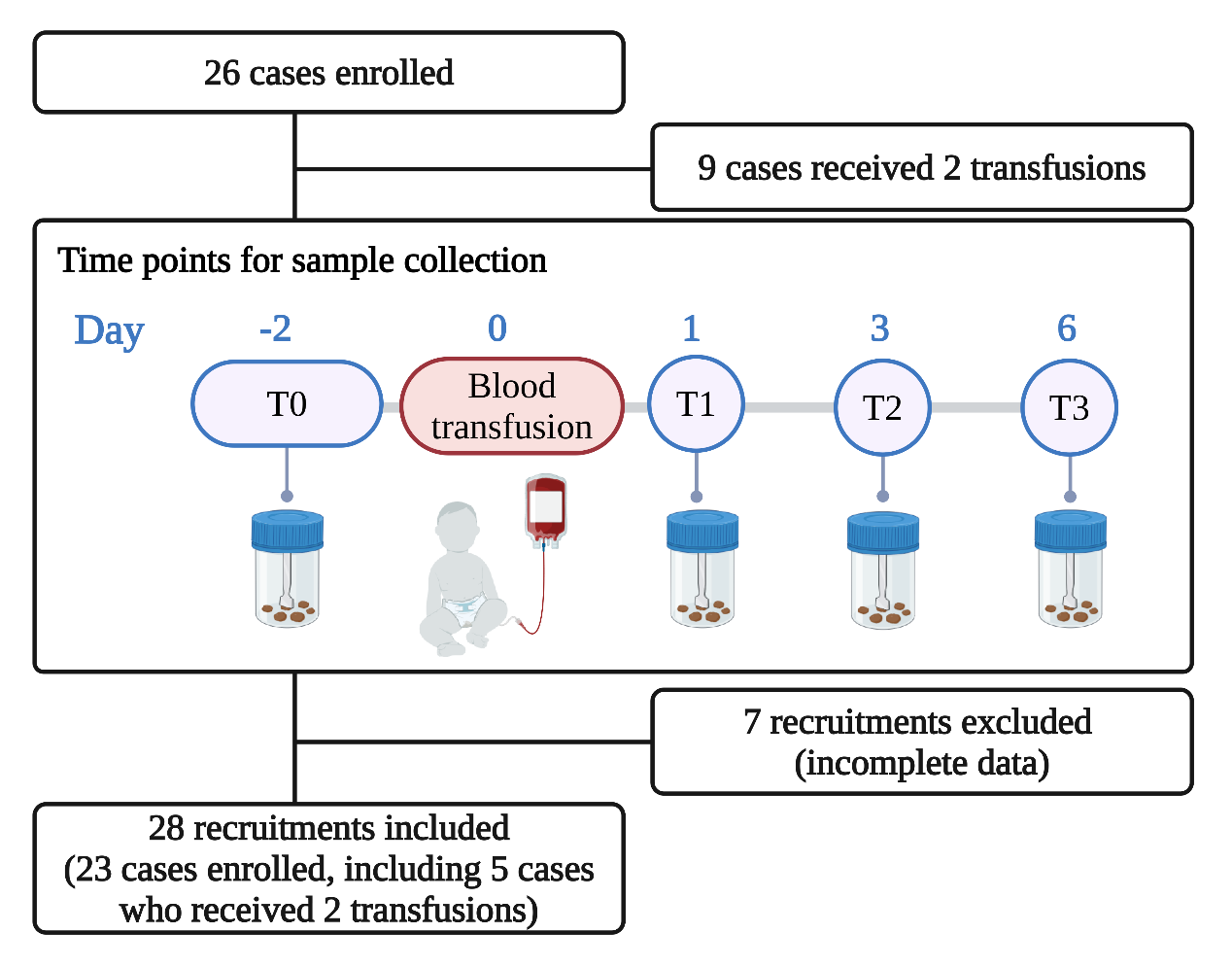
**

**Figure S1** Flow chart of the recruitment process of preterm infants. Mothers were invited to enroll in this study if they had given birth to preterm infants. When preterm infants were transfused with blood due to medical indications, fecal samples were collected at three time points: 1-2 days prior to the transfusion (designated as T0), then on the 1st, 3rd, and 6th day post-transfusion (designated as T1, T2 and T3 respectively). The fecal samples were then subjected to 16S rRNA gene-based NGS sequencing to characterize the gut microbial composition. (Created with BioRender.com).

**
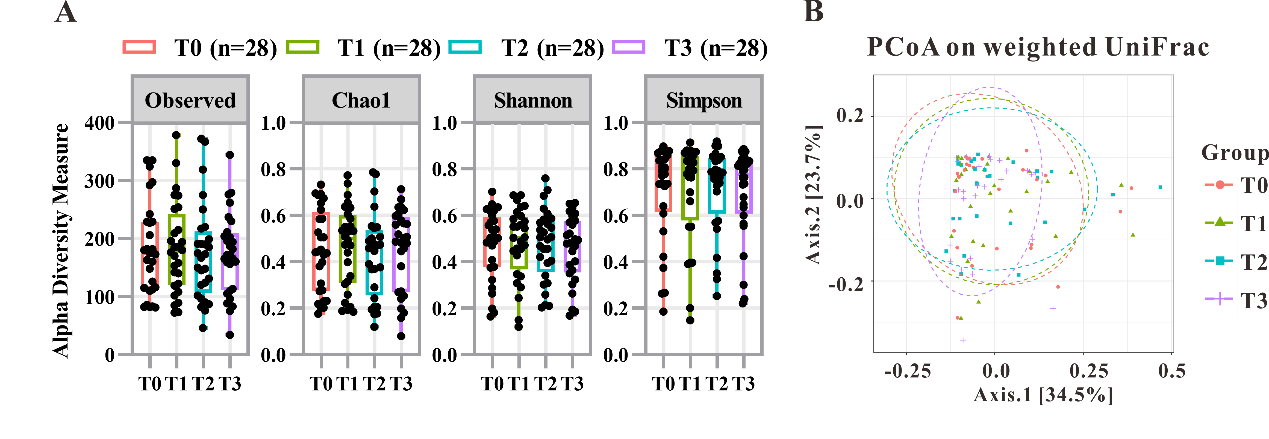
**

**Figure S2** Gut microbial analysis before and after blood transfusion. Fecal samples from 28 recruitment events at T0, T1, T2 and T3 were subjected to 16S rRNA gene-based NGS analysis. The bacterial communities were analyzed using (A) alpha-diversity and (B) beta-diversity.

**Supplementary Table 1** Characteristics of the study population.

| **Preterm infants** | **N = 23 (Mean ± SD or N)** |
| --- | --- |
| Birth weight (g) | 1370.7 ± 737.4 |
| Gestational age at birth (weeks) | 29.6 ± 3.3 |
| Gender, Male (N) | 15 |
| Vaginal delivery (N) | 10 |
| **Recruitment events** | **N = 28 (Mean ± SD)** |
| Body weight at the time of transfusion (g) | 2019.6 ± 881.3 |
| PMA at the time of transfusion (day) | 246.3 ± 31.1 |
| DOL at the time of transfusion (day) | 42.8 ± 30.7 |

**Supplementary Table 2** Calculated DEHP exposure doses in study subjects.

| **Blood bag** | **N = 10 (Mean ± SD)** |
| --- | --- |
| DEHP level in blood bag (mg/mL) | 0.072 ± 0.03 |
| DEHP dose per transfusion (mg/kg BW) | 1.44 ± 0.6 |

**Supplementary references**

1. Yang YS, Chang HW, Lin IH, Chien LN, Wu MJ, Liu YR, et al. Long-term Proton Pump Inhibitor Administration Caused Physiological and Microbiota Changes in Rats. Sci Rep. 2020;10(1):866.

2. Callahan BJ, Sankaran K, Fukuyama JA, McMurdie PJ, Holmes SP. Bioconductor Workflow for Microbiome Data Analysis: from raw reads to community analyses. F1000Res. 2016;5:1492.

3. Quast C, Pruesse E, Yilmaz P, Gerken J, Schweer T, Yarza P, et al. The SILVA ribosomal RNA gene database project: improved data processing and web-based tools. Nucleic Acids Res. 2013;41(Database issue):D590-6.

4. Schliep KP. Phangorn: phylogenetic analysis in R. Bioinformatics. 2011;27(4):592-3.

5. McMurdie PJ, Holmes S. phyloseq: an R package for reproducible interactive analysis and graphics of microbiome census data. PLoS One. 2013;8(4):e61217.

6. Chen J, Bittinger K, Charlson ES, Hoffmann C, Lewis J, Wu GD, et al. Associating microbiome composition with environmental covariates using generalized UniFrac distances. Bioinformatics. 2012;28(16):2106-13.

7. Segata N, Izard J, Waldron L, Gevers D, Miropolsky L, Garrett WS, et al. Metagenomic biomarker discovery and explanation. Genome Biol. 2011;12(6):R60.

8. Asnicar F, Weingart G, Tickle TL, Huttenhower C, Segata N. Compact graphical representation of phylogenetic data and metadata with GraPhlAn. PeerJ. 2015;3:e1029.
